# Association between neutropenia and IgG antineutrophil antibodies in a case of *CD40LG* deficiency due to two novel mutations

**DOI:** 10.1002/ccr3.2621

**Published:** 2019-12-25

**Authors:** Kristian Assing, Kaspar René Nielsen, Helene Broch Tenstad, Marianne Antonius Jakobsen, Christian Nielsen, Dorthe Grosen, Ulla Birgitte Hartling

**Affiliations:** ^1^ Department of Clinical Immunology Odense University Hospital Odense Denmark; ^2^ Department of Clinical Immunology Aalborg University Hospital Aalborg Denmark; ^3^ Hans Christian Andersen’s Children Hospital Odense University Hospital Odense Denmark; ^4^ Present address: Department of Pediatrics Aarhus University Hospital Aarhus Denmark

**Keywords:** CD40 ligand deficiency, hyper‐IgM syndrome, neutropenia, neutrophil IgG auto‐antibodies

## Abstract

This case suggests a mechanistic rationale for the clinical efficacy of intravenous immunoglobulins (IVIG) in treating CD40 ligand (CD40L) deficiency associated neutropenia as it is the first reported instance of free and cell‐bound antineutrophil antibodies in a case of CD40L deficiency, accompanied by a prolonged and clinically severe neutropenia.

## Graphical abstract

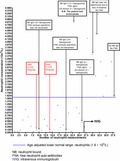

## 1 INTRODUCTION

The CD40 ligand (CD40L) expressed by *CD40LG* fulfills diverse functions. T cell‐expressed CD40L is involved in the stimulation of germinal center B cells, antibody isotype switching and the generation of high‐affinity antibodies through the process called somatic hypermutation (SHM). Functional mutations in *CD40LG* result in a hyper‐IgM syndrome characterized by normal to elevated IgM levels and low to absent IgG and IgA levels.

## 2 CASE PRESENTATION

**Figure 1:**
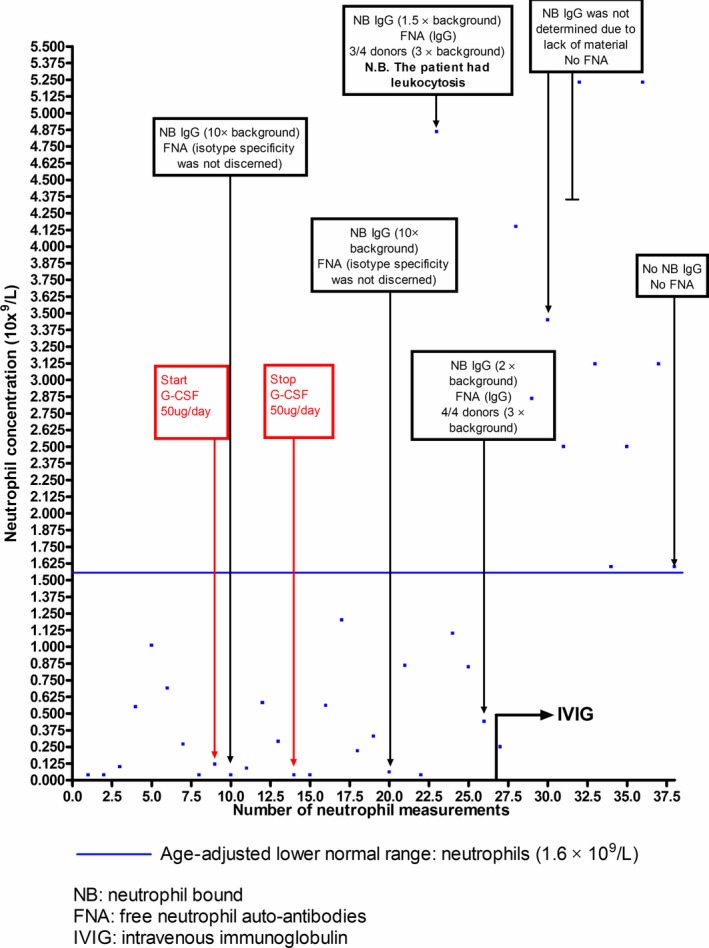
The patient's neutrophil concentrations during 28 mo (with 15 mo of neutropenia) divided upon 38 measurements. The blue line depicts the age‐adjusted neutrophil concentration (lower normal range). G‐CSF (50 μg/d) was administered daily for 7 d. Ten grams of intravenous Privigen^®^ were administered every fourth week

A 12 months old male patient was referred to our university hospital due to a respiratory infection. Neutropenia, which subsequently lasted for 15 months (with neutrophil concentrations often <0.05 × 10^9^/L (neutropenia grade 4), Figure 1), was diagnosed. The patient was not thrombocytopenic and his erythrocytes were direct antiglobulin test (DAT) negative. Antinuclear antibodies (ANA) were not detected. He received subcutaneous granulocyte‐colony stimulating factor (G‐CSF), according to recommended dosage^1^ (5 μg/kg/day = 50 μg Neupogen^®^/day), for seven consecutive days, without effect on his neutrophil levels but for a marked increase in his eosinophil counts: 2.2 × 10^9^/L (age‐adjusted range: <0.05 × 10^9^/L). A bone marrow biopsy showed lively erythropoiesis, normal thrombocytopoiesis, inhibited neutropoiesis, normal eosinophilopoiesis, and no signs of neoplastic hematologic disease. A possible cellulitis, located to his left instep, responded to amoxicillin and clavulanic acid. After discharge from the hospital, he was given preventive amoxicillin. In the following 10 months, his main complaints were gingivitis, oral ulcers, and mucocutaneous candidiasis, the latter amenable to oral antifungals. Next‐generation sequencing, using a custom made in‐house panel (Thermo Fischer Scientific) found no mutations in genes associated with severe congenital neutropenia: *ELANE, HAX1, GFI1, WAS, or G6PC3* but revealed that the patient was hemizygous for two new genetic variants in *CD40LG*: LRG_141, c.[**517G > C**; **521delA**] p.(Ala173Pro; Gln174fs). The patient's two *CD40LG* variants were confirmed by Sanger sequencing. None of the patient's *CD40LG* variants were described in the dbSNP database at NCBI, nor in HGMD, nor in Exome Aggregation Consortium nor in 1000 Genomes Data. Three (SIFT, PolyPhen‐2, and mutationassessor.org) out of six prediction tools rated the missense variant c. 517G > C as probably damaging. The other deletion variant c. 521delA, which caused a frameshift at amino acid position 174 (Gln174fs) and introduced a stop codon at amino acid position 190, was clearly damaging. According to the sequence variant nomenclature: varnomen.hgvs.org, the two *CD40LG* variants are to be described as separate mutations.

The same *CD40LG* variants (in cis) were detected by Sanger sequencing in the patient's mother. The patient had undetectable IgG (<0.4 g/L), IgA (<0.05 g/L) but elevated IgM (8.45 g/L) levels consistent with the hyper‐IgM syndrome. A lymphocyte marker study, performed at the age of 27 months, showed normal CD4^+^, CD8^+^ T, and CD19^+^ B‐cell concentrations but total absence (0%) of isotype switched (CD27^+^IgD^−^) memory B cells (age adjusted 5‐95 percentile range: 4.7%‐21.2% of CD19^+^ B cells). Patient B cells exhibited reduced SHM (determined by a restriction enzyme‐based hot spot mutation assay [REHMA]) of kappa light chain genes: 2% (age‐adjusted normal range: >10%, Tissue Typing laboratory, National University Hospital, Copenhagen). *Clostridium tetani* antibody levels (following three tetanus toxoid vaccinations) were nonprotective (<0.01 IU/mL, Statens Serum Institut). Ex vivo stimulation of his CD3^+^ T cells, with phorbol 12‐myristate 13‐acetate (PMA: 20 ng/mL) and ionomycin (300 ng/mL), failed to upregulate surface CD40L, although his CD3^+^ T cells were activated as evidenced by upregulation of surface CD69. Repeated testing of patient sera by the Luminex‐based LABScreen multi‐assay (prior to institution of antibody replacement therapy) revealed no antibody specificities toward a wide range of human leukocyte antigen (HLA)‐class I, HLA‐class II determinants as well as toward human neutrophil antigens (HNAs). Patient sera (prior to and after institution of antibody replacement therapy) and patient neutrophils were tested for granulocyte‐reactive antibodies using flow direct‐granulocyte immunofluorescence test (D‐GIFT), flow indirect‐granulocyte immunofluorescence test (I‐GIFT), granulocyte agglutinations tests (GAT), and monoclonal‐antibody‐specific immobilization of granulocyte antigens (MAIGA) as earlier described.^2^ Results were confirmed by another ISBT granulocyte immunology reference laboratory (Karolinska University Hospital). Patient neutrophils were repeatedly (moderately to heavily) covered with IgG (Figure 1) and the corresponding patient sera contained free neutrophil reactive (IgG) antibodies (Figure1). Possibly reflecting the aftermath of reactive leukocytosis (concomitant leukocyte: 20.5 × 10^9^/L (normal range <14.0 × 10^9^/L) and lymphocyte concentration: 13.8 × 10^9^/L (normal range <7.9 × 10^9^/L), but with normal CRP (<1 mg/L), we recorded a single instance where the patient's neutrophil concentration was normalized (≥1.6 × 10^9^/L), (Figure 1) despite the presence of neutrophil auto‐antibodies. The more prolonged normalization of our patient's neutrophil counts coincided with the disappearance of free and cell‐bound neutrophil antibodies (measured on three different occasions, Figure1). While on IVIG (10 g Privigen^®^/4th week was initiated at the age of 27 months, Figure 1) and prophylactic sulfamethoxazole with trimethoprim, our patient, aged three years and 6 months, underwent successful allogeneic bone marrow transplantation. Written consent by the patient's parents and permission from the chairman of the Regional Committee on Health Research Ethics for Southern Denmark (case S‐20192000‐48) was obtained.

## 3 DISCUSSION

Neutropenia affects approximately two‐thirds of patients with CD40L deficiency, leading to significant comorbidity due to mucocutaneous inflammation.^3^ The pathogenesis underlying CD40L deficiency associated neutropenia has not yet been clearly delineated. As bone marrow stromal cells express CD40,^4^ stimulation by CD40L can induce them to produce G‐CSF.^5^ Hence, CD40L deficiency could compromise the ability of bone marrow stromal cells to secrete G‐CSF, thereby causing neutropenia. Supporting that hypothesis, treatment with G‐CSF has been reported to increase or normalize the neutrophil counts in CD40L deficiency.^6^ However, seven days of treatment with a standard dosage of G‐CSF did not affect our patient's neutropenia but instead markedly increased his peripheral eosinophil concentration, indicating that the G‐CSF levels were sufficient to induce myelopoietic lineage differentiation. Still, we cannot exclude that the standard G‐CSF dosage was insufficient, since the patient, despite the presence of antineutrophil antibodies, experienced a pronounced, possibly reactive, neutrophil increase, consistent with G‐CSF responsiveness. Infection‐induced G‐CSF secretion results in massive bone marrow neutrophil release^7^ which may override the effects of neutrophil antibodies. Hence, pronounced bone marrow release of neutrophils, secondary to infection, may have effected our patient's transiently normalized neutrophil count in the presence of neutrophil auto‐antibodies. Theoretically, our patient could have experienced additional unrecorded instances of normalized neutrophil concentrations, however, his prolonged clinical complaints, involving gingivitis, oral ulcers, and mucocutaneous candidiasis, indicated sustained neutropenia.

The clinical efficacy of IVIG, in treating CD40L deficiency associated neutropenia,^8^ suggests that autoimmune mechanisms might also be involved, although autoimmune neutropenia has never been confirmed in this disorder^8^ and some authors even mention it as unlikely.^9^ We hereby represent the first case of CD40L deficiency, accompanied by undetectable plasma IgG and IgA concentrations, but where free and cell‐bound antineutrophil IgG antibodies coincided with a protracted, clinically significant neutropenia. We were not able to determine the subclass specificity of these IgG antibodies, but to varying degrees all IgG subclasses may engage Fc‐receptors and thereby contribute to splenic opsonization of antibody‐coated neutrophils. The mechanism (s) underlying our patient's sparse production of IgG is uncertain. However, CD40L independent production of IgG toward viral glycoproteins has been documented^10^ and neutrophils express several surface glycoproteins, such as CD11b^11^ and CD47,^12^ which could constitute targets for CD40L independent IgG production. Interestingly, Litinskiy et al^13^ have demonstrated that human dendritic cells can induce CD40L independent IgG class shift through expression of the B‐cell stimulator proteins: B‐cell activating factor of the tumor‐necrosis‐factor family (BAFF) and a proliferation‐inducing ligand (APRIL). We were not able to confirm the antigen specificity of the IgG antibodies, however, we cannot preclude that the antibodies were “neutrophil specific” as other autoantigen specificities were not detected (no thrombocytopenia, the patient was DAT negative and we observed no ANA reactivity). However, the lack of any known neutrophil antigen specificity of these antibodies concurred with our patient's severely reduced SHM, as CD40L is crucial for SHM. Auto‐reactivity is often a feature of compromised SHM, since inherently auto‐reactive B‐cell clones are prevented from rescuing themselves, through the process of SHM, from auto‐reactivity, a process called “clonal redemption”.^14^

## 4 CONCLUSION

We present a case of CD40L deficiency where the presence of neutrophil reactive IgG coincided with a clinically severe neutropenia. Due to its diverse immunomodulatory capabilities, IVIG therapy is widely used for treating autoimmune disorders. Our case suggests a mechanistic rationale for the clinical efficacy of IVIG in attenuating CD40L deficiency associated neutropenia and substantiates models showing that the requirement for CD40L, in mediating B‐cell IgG isotype switching, can be circumvented.

## CONFLICT OF INTEREST

The authors have no conflicts of interest relevant to this article to disclose.

## AUTHOR CONTRIBUTIONS

Kristian Assing conceptualized the case, collected blood samples and wrote the final paper. Kaspar René Nielsen performed the neutrophil specific auto‐antibody assays and critically read the final paper. Helene Broch Tenstad collected blood samples, wrote parts of the initial paper and critically read the final paper. Marianne Antonius Jakobsen performed genetic work‐up and critically read the final paper. Christian Nielsen performed flow‐cytometric analyses and critically read the final paper. Dorthe Grosen organized blood sampling, attended the patient and critically read the final paper. Ulla Birgitte Hartling attended the patient, collected blood samples, wrote parts of the initial paper and critically read the final paper. All authors approved the final manuscript as submitted and agree to be accountable for all aspects of the work.
